# Case report: acute pancreatitis caused by postcholecystectomic hemobilia

**DOI:** 10.1186/1471-230X-10-75

**Published:** 2010-07-07

**Authors:** Halil Alis, Mehmet A Bozkurt, Osman Z Oner, Kemal Dolay, Ahmet N Turhan, Adem Uçar, Ercan Inci, Ersan Aygun

**Affiliations:** 1 Department of General Surgery, Bakirkoy Dr. Sadi Konuk Train And Research Hospital, Tevfik Saglam Road, Istanbul, Turkey; 2 Department of Radiology, Bakirkoy Dr. Sadi Konuk Train And Research Hospital, Tevfik Saglam Road, Istanbul, Turkey; 3 Department of Interventional Radiology, Istanbul University Istanbul Faculty of Medicine, Capa, Istanbul, Turkey

## Abstract

**Background:**

Hemobilia is a rare cause of upper GI bleeding and the reasons for the majority of the cases are iatrogenic. It is also one of the rarest vascular complication following laparoscopic cholecystectomy but acute pancreatitis due to postcholecystectomic hemobilia as a late complication of cholecystectomy is not yet described.

**Case Presentation:**

We presented the case of a 32-year-old female, admitted to our emergency surgery clinic with hematemesis, jaundice and abdominal pain who had a history of laparoscopic cholecystectomy 4 months ago. Patient was diagnosed as acute pancreatitis and obstructive jaundice caused by postcholecystectomic hemobilia. Afterwards she is successfully treated by ERCP, angiographic identification and embolization of right hepatic artery pseudoaneurysm.

**Conclusions:**

We presented that postcholecystectomic hemobilia may cause acute pancreatitis and acute pancreatitis caused by postcholecystectomic hemobilia should also be included to the rare complications which may occur following cholecystectomy.

## Background

Hemobilia is a rare cause of upper GI bleeding and the reasons for the majority of the cases are iatrogenic. It is also one of the rarest vascular complication following laparoscopic cholecystectomy but acute pancreatitis due to postcholecystectomic hemobilia as a late complication of cholecystectomy is not yet described.

We report a case presented with acute pancreatitis caused by the hemobilia as a complication of cholecystectomy.

## Case Presentation

A 32-year-old female presented with complaints of abdominal pain and hematemesis. History revealed that she has been suffering severe right upper quadrant pain for one year which resolved 4 months ago following elective laparoscopic cholecystectomy. Cystic artery and cystic duct was explored during operation and cholecystectomy was completed without any difficulty. Her physical examination revealed jaundice, abdominal tenderness, and melena. Her heart rate was 98/min., blood pressure 100/60 mm/hg and body temperature 36.7 C. Her abnormal blood analysis results were as following; hemoglobin: 6.4 g/dl, hematocrite: 21%, MCV: 70 fl, AST: 435 IU/L, ALT: 220 IU/L, GGT: 256 IU/L, LDH: 400 IU/L, amylase: 2046 IU/L, lipase: 7339 IU/L, total bilirubine: 8.0 mg/dl and direct bilirubine: 6.2 mg/dl.

**Figure 1:**
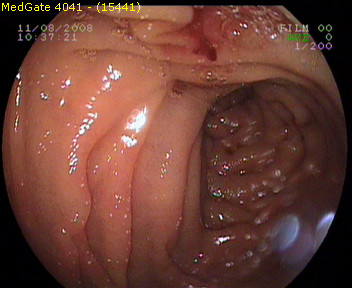
**initial endoscopy demonstrating blood leakage through duodenal papilla**.

Nasogastric intubation and irrigation confirmed the presence of upper GI bleeding. Ten hours after admission source of the bleeding was identified as duodenal papilla by the emergency upper GI endoscopy where esophagus, stomach and bulbus were found to be normal (Figure 1: Initial endoscopy demonstrating blood leakage through duodenal papilla).

**Figure 2:**
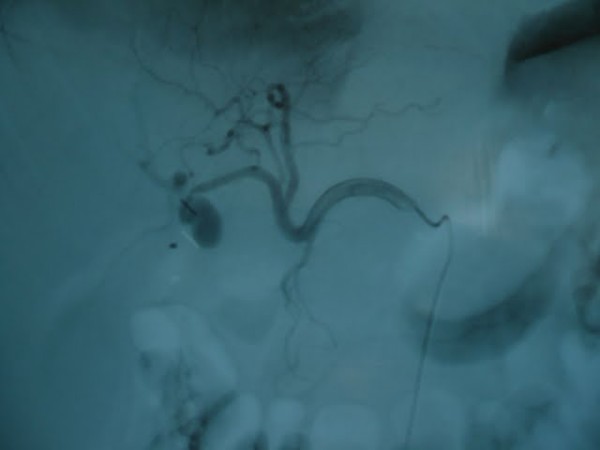
**pseudoaneurysm**.

**Figure 3:**
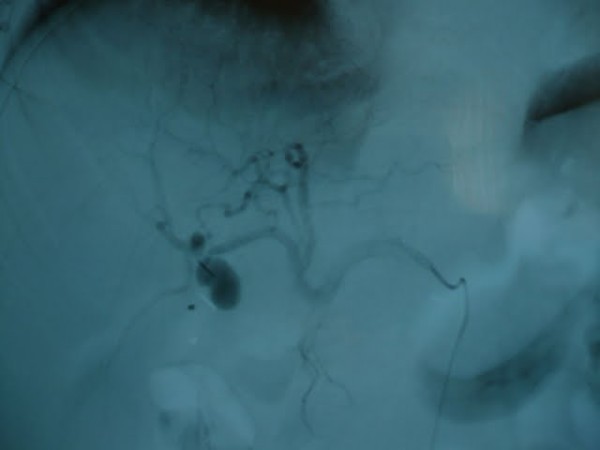
**pseudoaneurysm**.

Abdominal ultrasonography did not demonstrate any pathological finding except confirming that patient was cholecystectomised. Abdominal computerized tomography demonstrated a dimensions were 2 × 1.8 cm at portal hilus and it was connected to the right portal vein with a fenestration in diameter of 5 mm. (Figure 2 and figure 3: pseudoaneurysm) During diagnostic evaluation period, intravenous fluid resuscitation was made and hemoglobin level of the patient was brought to 9.6 g/dl by 3 units of erythrocyte suspension transfusion.

ERCP was performed with the diagnose of acute pancreatitis caused by hemobilia at the 16^th ^hour after the admission. Ongoing blood leak through papilla was visualized. Contrast serial graphies after canulation of the papilla revealed a dilated common bile duct with a diameter of 18 mm and mobile filling defects with labile shapes suggesting a thrombus. We supposed that the dilatation in the common bile duct was a chronic process. A sphincterotomy, 10 mm in length was made. Thrombus was removed with balloon catheter. The amount of ongoing hemorrhage after ERCP was not much to cause an alarm, patient was rapidly referred to angiography unit for further diagnose and treatment.

**Figure 4:**
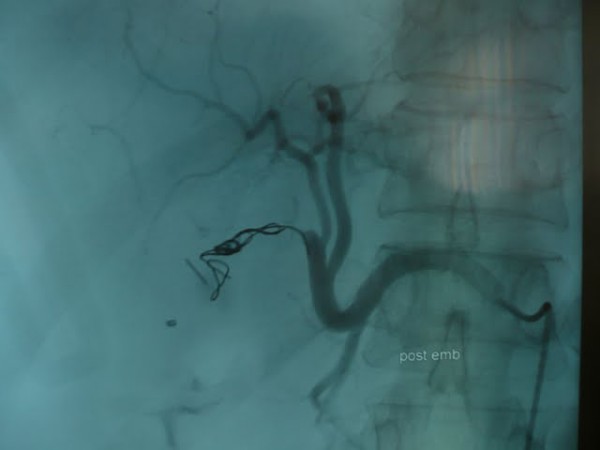
**right hepatic artery after embolization**.

**Figure 5:**
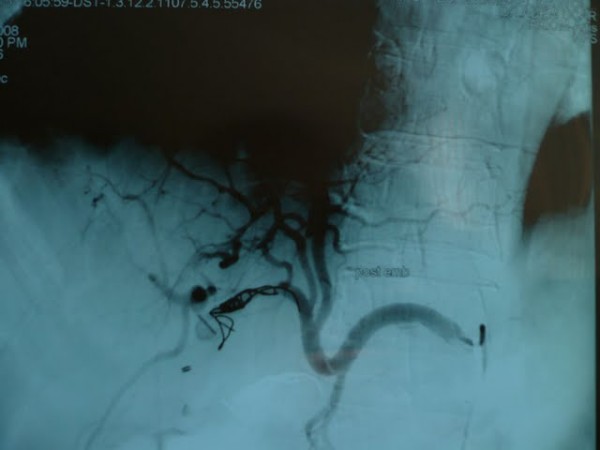
**right hepatic artery after embolization**.

Selective angiography revealed a pseudoaneurysm of right hepatic artery connected to remaining cystic duct and angioembolization was made during the same intervention (Figure 4 and figure 5: right hepatic artery after embolization). Hemorrhage was stopped after the angioembolization.

After ERCP and angioembolization, patient's abnormal amylase, lipase and liver function test results begun to decrease rapidly to the normal values. No additional blood transfusion was required. Oral feeding was allowed on the second day and the patient was discharged on the fourth day of the intervention.

## Discussion

The term hemobilia was first used by Sandblom to describe bleeding into the biliary system after a subcapsular liver injury but this therm now covers all reasons which causes bleeding in to the biliary three [1]. It is an uncommon cause of upper gastrointestinal hemorrhage but the widespread use of invasive hepatobiliary procedures and improved recognition has increased its incidence [2]. Severe hemobilia is considered with hemorrhage resulting in hemodynamic instability or necessitating transfusion [3]. In the majority of cases the cause is iatrogenic (liver biopsy, percutaneous transhepatic cholangiography [2]. Tumors, inflammatory disorders, and gallstones, vascular diseases such as aneurysm and bleeding disorders may also cause hemobilia [4]. Among 55 well-documented cases of hemobilia, 53% were originated in the liver, 23% in the gallbladder, 22% in the bile ducts, and 2% in the pancreas [5]. Less common causes are pseudoaneurysm of the hepatic artery [6].

Clinical presentation of hemobilia can be variable and intermittent [1]. Hemobilia classically presents as biliary colic, jaundice, hematemesis, and melena. However, most patients do not present with all four of these findings [2]. Blood may clot in various locations depending on the amount and rate of bleeding, hematemesis or melena may occur [7]. If blood clots within the bile duct, it may cause obstructive jaundice or pancreatitis [4]. Acute biliary symptoms and signs combined with acute upper gastrointestinal bleeding should strongly suggest the possibility of hemobilia [4].

For the diagnosis and evaluation of hemobilia sonography and computed tomography are helpful in assessing the possibility of tumor or stone disease [5].

Magnetic resonance imaging can distinguish blood from stones and sludge and may be helpful when the diagnosis is uncertain [8]. The diagnosis of hemobilia is most frequently made by upper endoscopy or ERCP [9]. If bile duct obstruction is suspected, early ERCP is advisable [7]. Angiography should be considered early in cases of trauma or known tumor for both diagnosis and therapy; it is useful in localizing the site of bleeding [4].

Hemobilia from a hepatic artery source frequently requires transcatheter embolization via a right femoral artery approach or even hepatic artery ligation, which can result in liver failure in patients with underlying hepatic insufficiency [3]. Angiography may provide an opportunity for arterial embolization which can be a simple, safe, and effective treatment for hemobilia with less morbidity and mortality than surgery [8].

Vascular injuries during laparoscopic cholecystectomy may occur in 0.2%-0.5% cases and these injuries are also reported that they may cause hemobilia with pseudoaneurysmatic dilatation [4]. Postcholecystectomic hemobilia usually occurs as a result of cystic or right hepatic arterial wall injury [8]. Injury of common bile duct wall is also thought to be able to cause hemobilia [8]. Even if it is not yet proved, vascular wall injury may be caused by physical, thermal and chemical injury during laparoscopy [4]. Furthermore operation reports of the hemobilia patients frequently describe inflamed gallbladders and difficult cholecystectomies [4].

Postcholecystectomic hemobilia usually occurs 4 weeks after operation [10]. In case of massive bleeding mortality rate can be as high as 50% [11]. Blood clot may cause common bile duct occlusion which may lead to jaundice and pancreatitis as in other causes of hemobilia [3].

## Conclusions

Hemobilia after cholecystectomy and acute pancreatitis after hemobilia which resulted from liver trauma are previously and separately described conditions in the literature. But hemobilia rising from a pseudoaneurysm after cholecystectomy and causing acute pancreatitis was not yet described. Our patient had a hemobilia which caused obstructive jaundice and acute pancreatitis 4 months after cholecystectomy.

We treated her as it is suggested in the literature with ERCP and angiographical embolization having a successful outcome. The special point which we would like to underline is that we defined a new late complication of cholecystectomy; postcholecystectomic pseudoaneurysmatic hemobilia causing acute pancreatitis.

## Competing interests

The authors declare that they have no competing interests.

## Authors' contributions

EA, HA and MAB undertook the management of our patient from the time of his initial presentation to ERCP and follow up examination. OZO and ANT performed the endoscopy, KD and EA performed the ERCP. AU and EI achieved the angiography and CT. All authors read and approved the final manuscript.

## Consent

Written informed consent was obtained from our patient for publication of this case report and any accompanying images. A copy of the written consent is available for review by the Editor-in-Chief of this journal.

## Pre-publication history

The pre-publication history for this paper can be accessed here:

http://www.biomedcentral.com/1471-230X/10/75/prepub
